# Environmental Insights into Single-Cell Protein Production: A Life Cycle Assessment Framework

**DOI:** 10.1021/acssuschemeng.5c02336

**Published:** 2025-05-31

**Authors:** Eva Martínez-Ibáñez, Jara Laso, Marta María Pérez-Martínez, Raquel Martínez-Vazquez, David Baptista de Sousa, Diego Méndez, Elena Olaya-Pérez, Virginia Marchisio, Ruben Aldaco, María Margallo

**Affiliations:** † Department of Chemical and Biomolecular Engineering, 16761 University of Cantabria, Av. Los Castros s/n, Santander, Cantabria 39005, Spain; ‡ 661049 EnergyLab Technology Center, Fonte das Abelleiras s/n, Vigo, Galicia 36310, Spain; § ANFACO-CECOPESCA, Department of Sustainability and Circular Economy, Campus University 16, Vigo, Galicia 36310, Spain

**Keywords:** single cell protein, LCA, scale-up, environmental impacts, food waste

## Abstract

Innovative protein sources, such as single cell protein (SCP) derived from unicellular organism biomass, are emerging as promising solutions to address food scarcity and meet global nutritional needs. This article aims to estimate the environmental impacts of SCP production using biomethane from fish industry waste through an ex-ante Life Cycle Assessment (LCA), focusing on scaling up a lab-scale process. The proposed scenarios include SCP production with biofertilizer recovery (baseline scenario) and the additional valorization of biomethane as grid gas, electricity, and/or heat (modified scenarios). The analysis follows a cradle-to-gate approach, and recovered materials and energy were included by expanding the system boundaries to account for avoided primary production. Results revealed significant differences between laboratory-scale and industrial-scale impacts, with reductions ranging from 60% to 96% across all impact categories when scaled up. Focusing on the industrial scale, the baseline scenario showed the poorest environmental performance, mainly due to biogenic methane emissions from unutilized biogas. In contrast, modified scenarios that incorporated various biomethane utilization pathways achieved substantial reductions across all impact categories. These findings suggest that the optimal system configuration combines the recovery of biomethane, heat, and electricity, underscoring the need for further research into its technical and economic feasibility within the food sector. This research highlights the utility of LCA in evaluating emerging technologies, identifying key environmental challenges, and guiding decision-making at early development stages.

## Graphical abstract

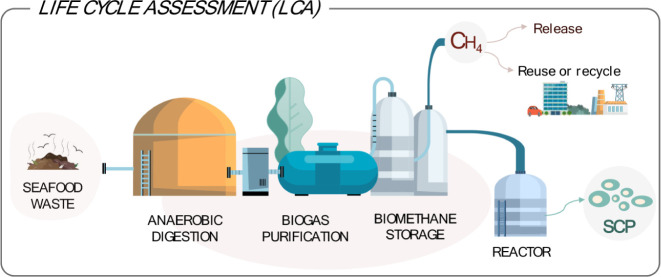

## Introduction

The production of high-quality food, accessible to the entire population and whose production leads to a reduction in environmental impacts, is essential to achieving sustainable development goals (SDG) 2 “Zero Hunger” and 12 “Responsible consumption and production”.^1^ This challenge becomes even more pressing in light of the projected global population growth, which is expected to reach 10 billion by 2050, significantly increasing the demand for nutritious and sustainable food sources.^2^ Meeting this demand requires transformative changes in food systems to keep resource use and emissions within planetary boundaries,^3^ which must be respected to prevent significant and potentially irreversible changes to the Earth’s system.^4^ Circular economy-based practices and technologies are crucial for decoupling food production from resource depletion while providing quality protein sources.^5^

To address food scarcity concern, novel foods such as microbial products, like single cell proteins (SCPs), are gaining increasing interest within the scientific community due to their potential to reduce the environmental impacts of the food system, while meeting nutritional needs.^6^ SCPs refers to proteins derived from unicellular microorganisms including bacteria, yeasts, algae, and fungi,^7^ which break down complex substrates into simpler compounds for their synthesis.^8^ In addition to its high protein content (60–82% dry matter), SCP contains carbohydrates, nucleic acids, fats, vitamins, and essential amino acids that are not present in other animal and plant sources.^9^ This emerging protein source presents a promising solution to the global shortage of protein-rich foods, being considered as a substitute for animal- and plant-based proteins for both human and animal consumption.^10^

Microbial protein has traditionally been produced using fossil resources such as natural gas.^11^ Current research has evolved toward valorizing waste derived from agricultural or industrial sources, also known as second-generation materials, as substrates for cultivating protein-rich microorganisms.^12^ This approach offers a dual advantage: the valorization of streams that would otherwise be discarded (e.g., wastewater streams, agricultural waste, or industrial byproducts among others), promoting the evolving circular economy,^7^ and the efficient and sustainable production of high-quality protein.^13^ The use of industrial or agricultural residual streams as substrate for producing SCP biomass tends to reduce some environmental impacts, such as air emissions, soil contamination, waste generation, or use of natural resources, compared to other conventional carbon sources. However, these claims about the environmental sustainability of SCP production with byproducts can only be supported by objective methodologies that allow for the measurement of environmental impacts.^14^ Life Cycle Assessment (LCA) provides a holistic approach to assess the environmental impacts of a product, process, or service throughout its entire life cycle.^15^ It is a widespread methodology for making decisions about a system addressing challenges and needs.^16^ Hence, several studies can be found in the literature applying LCA to a variety of substrates to cultivate SCP, like crude pea starch,^17^ oat side stream,^12^ rice straw,^18^ wheat grain,^19^ or wastewater from potato industry^20^ among other substrates. However, to the best of the authors’ knowledge, no LCA study has explored the environmental impact of SCP production using byproducts of fish processing.

According to the literature, ex-ante or prospective LCA is defined as the analysis of future scenarios by scaling up emerging technologies to full-scale production and comparing them with established technologies.^20^ Following the recommendations of Ardvisson et al.,^21^ the term “ex-ante LCA” was used in the current study. This approach supports decision-making for new technologies before their commercial implementation, aiming to guide choices that ensure these technologies remain environmentally competitive compared to existing ones.^22^ However, it requires a shift from ex-post to ex-ante environmental assessment and introduces an additional challenge related to epistemic uncertainty, rising from the lack of case-specific data, which is a distinctive feature of future systems and models.^23^

Motivated by the gap of LCA studies on the valorization of fish industry byproducts, and the advantages of the ex-ante methodology in assessing the potential environmental impacts of emerging technologies at the laboratory scale, this study aims to perform a LCA of SCP biomass production using biomethane as a substrate, derived from the anaerobic digestion of fish processing byproduct. Due to the low maturity of the technology and the corresponding lack of primary industrial-scale data, the extrapolation of results to industrial scale remains limited in the bibliographic studies published so far.^6^ The statement of the novelty of this research focuses on scaling up using primary lab-scale data in order to preliminarily assess the critical sources of environmental impact in the process life cycle at a larger scale. Therefore, the findings of this study aim to determine whether SCP production is an environmentally viable solution for obtaining new protein sources that ensure environmental sustainability and food security.

## Methodology

In this study, the LCA methodology was employed to evaluate the environmental impacts of SCP production. The international standards UNE-EN ISO 14040 and 14044 were followed in detail for this purpose.^15,24^ The current study can be classified as a limited-scope ex-ante LCA, as it includes a technological scale-up (from low to high Technology Readiness Levels, TRL), but does not explicitly model projected changes in the background system, such as future energy mix, legislative developments, or global technological trends.

### Goal and Scope Definition

The current study aimed to evaluate, from an environmental perspective, the impacts and hotspots associated with SCP production using biomethane as a substrate, derived from the anaerobic digestion of fishery industry waste. The main function of the system was to produce an alternative protein source by utilizing biogas derived from fish industry waste. To quantify this function and relate all material and energy input and output flows of the system, the selected functional unit (FU) was defined as a mass-based unit: “1 kg of dried SCP to meet an increased demand for products intended for human consumption”.

As for the scope of the study, it was conducted from a cradle-to-gate approach (Figure 1). The process is a two-stage continuous system. In the first step, gaseous precursors were generated using a cosubstrate derived from byproducts of fish production, consisting of sludge (25%), process wastewater (50%), and homogenized surimi residues (25%) from a fish processing plant. The characteristics of the bio stream are summarized in Table S1. This stage involved the homogenization of waste streams, biogas production through anaerobic digestion (AD), storage and purification of the obtained gas, as well as the treatment and subsequent management of the digestate. The AD process is a well-established technology for energy recovery, particularly in the form of biomethane, from various agro-food residues and organic waste streams.^5^ Additionally, the digestate produced is commonly applied as an organic fertilizer. The second phase included the cultivation of microorganisms in a reactor equipped with a nutrient pumping system, biomass extraction, and protein recovery, as well as biomass conditioning through a centrifugation process to concentrate the SCP protein, followed by a drying process to remove the moisture.

**Figure 1:**
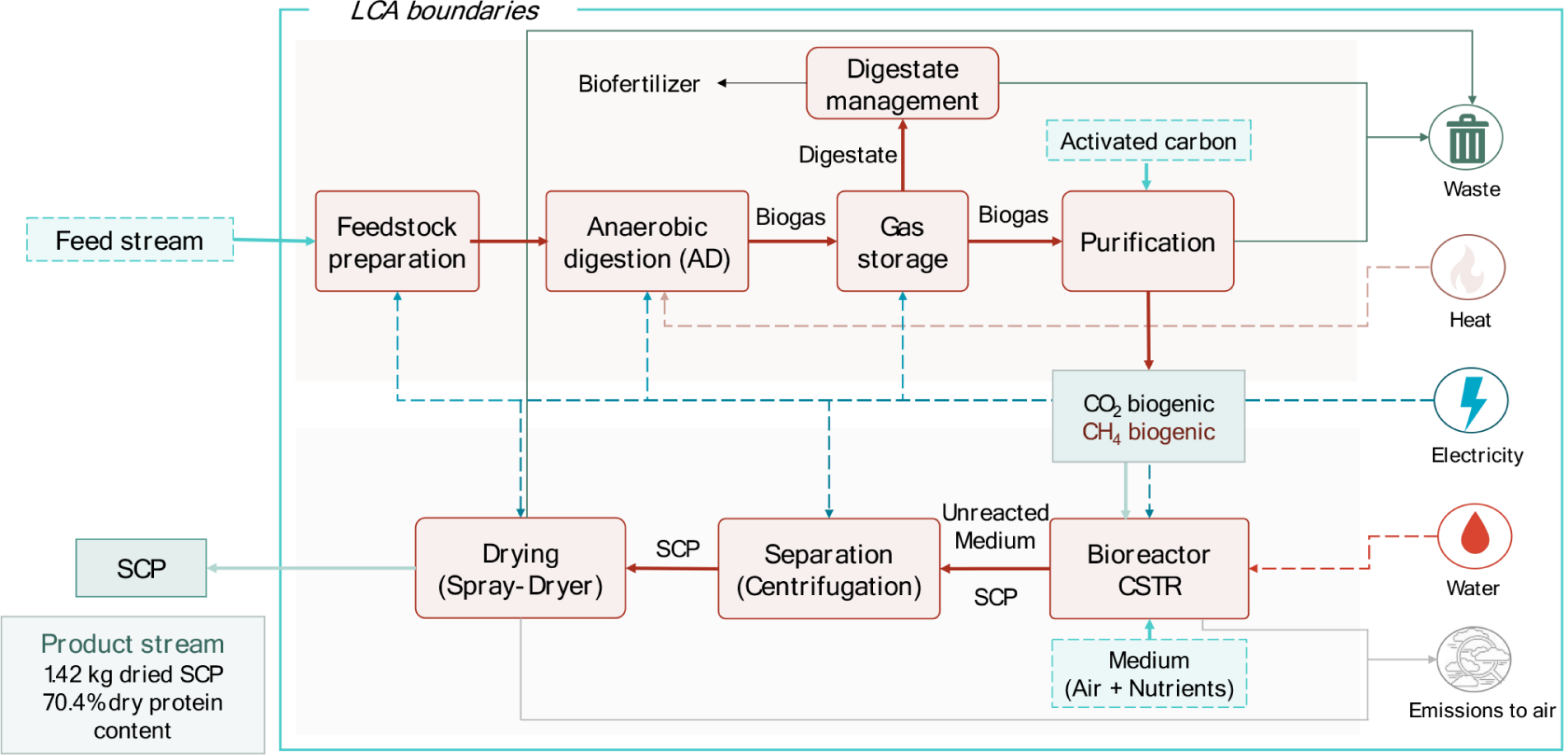
System boundaries of the baseline scenario that include two steps: biogas production using food industry waste, and microbial protein production using produced biogas. In the diagram, dashed lines and boxes represent the process inputs, while solid lines represent the process outputs.

Processes related to the construction and decommissioning phases of the plant, machinery, and industrial equipment were excluded, as these have been shown to have negligible environmental impacts compared to operational phases. Furthermore, it was assumed that all fish waste originated from a single processing facility colocated with the biogas production plant. This assumption eliminated the need to consider transportation impacts, which were deemed irrelevant to the study’s objectives and scope. During the biogas purification process, a CO_2_ stream was generated and released into the atmosphere. However, since that the carbon dioxide originated from biogenic sources, the emissions were not accounted in the analysis. The primary geographical focus was Spain, where the technical installations are situated. Consequently, the electricity consumed throughout the process was modeled based on the Spanish electricity production mix.^25^

The system boundaries included all flows of materials, chemicals, energy, and transportation involved in feedstock preparation, biomethane production, and single-cell protein production. Additionally, the system boundaries were expanded to include the processes displaced by the use of coproducts, such as compost, electricity, and/or heat, which were considered as avoided impacts from other markets. In other words, following the ISO guidelines, the system’s multifunctionality was addressed through system expansion; thus, the obtained coproducts were considered equivalent substitutes for commercial products.^26^

Experimental data collected at the laboratory scale constitute the first scenario analyzed (hereinafter referred to as the base case). These data were subsequently extrapolated to an industrial scale, as explained in the following section. At the industrial scale, the combination of assumptions regarding the utilization of coproducts from the SCP production plant led to the definition of four additional scenarios. A summary of the key characteristics of each scenario is provided in Table 1.

**Table 1:** Description of Each Scenario Evaluated in the Study

Scenario	Parameter	Description (Product and coproducts)
*Lab scale*		
Base case		Current production of SCP at lab scale (SCP-biofertilizer)
*Industrial scale*		
Baseline Sc.	-	Production of SCP by scaling up lab-scale data (SCP-biofertilizer)
Scenario 1	-	Baseline scenario, including the valorization of part of the biomethane as grid gas (SCP-biofertilizer-biomethane)
Scenario 2	-	Baseline scenario, assuming that the remaining biomethane from SCP production is used to cogenerate electricity and heat in a cogeneration heat and power (CHP) plant (SCP-biofertilizer-heat and electricity)
Scenario 3	-	Baseline scenario, assuming that the remaining biomethane from SCP production feeds a cogeneration plant for electricity and heat generation for internal process use (SCP-biofertilizer-heat and electricity for internal use)
1	Electricity	Influence of modifying the electricity supply in industrial-scale scenarios. Specifically, three electricity sources were considered: residual electricity, electricity with a guarantee of origin (renewable mix), and the current Spanish electricity grid mix.
*Sensitivity analysis*		
1	Electricity	Influence of modifying the electricity supply in industrial-scale scenarios. Specifically, three electricity sources were considered: residual electricity, electricity with a guarantee of origin (renewable mix), and the current Spanish electricity grid mix.
2	Allocation method	Influence of the allocation method used. In addition to system expansion, economic allocation and mass allocation were analyzed to handle the system’s multifunctionality

Figure 2 illustrates the system boundaries for the Baseline scenario and the three proposed scenarios, based on the different utilization pathways of the biomethane fraction not used for industrial-scale SCP production. In the Baseline scenario, SCP production involved the AD of fish production byproducts (biomass stream) in a reactor to produce biogas and digestate. The digestate was treated via centrifugation, and the solid fraction was valorized as a biofertilizer for field application, based on its nitrogen (N), phosphorus (P), and potassium (K) content. The biogas was upgraded to biomethane for SCP production, with the remaining fraction emitted into the atmosphere as biogenic methane.

**Figure 2:**
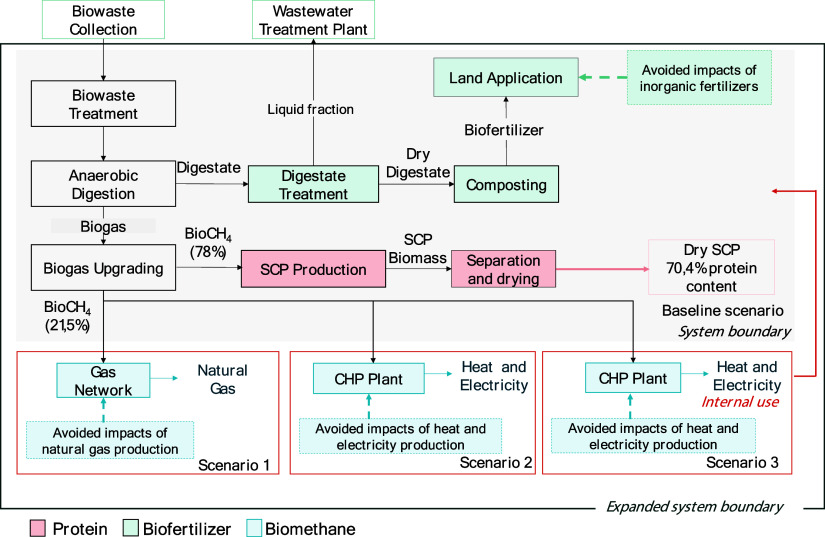
Process flow diagram and scenario description. The dashed line represents the avoided commercial products that were replaced by the biorefinery products.

In contrast, the modified scenarios propose the valorization of this biogenic methane stream into value-added products. Specifically, in Scenario 1, the remaining portion of bioCH_4_ was assumed to be utilized as grid gas instead of being emitted into the atmosphere. In Scenario 2, a feasible improvement was proposed in the form of combined heat and power generation using the remaining biomethane from SCP production. Finally, Scenario 3 explored the potential recirculation of energy obtained from the cogeneration plant for internal use, replacing energy supplied by external sources. In all scenarios, the system boundary was expanded to include marginal technologies for the substitution of products. This approach highlights the differences in system boundaries and the alternative pathways for valorizing the unused biomethane across the scenarios.

### Data Acquisition and Life Cycle Inventory

The life cycle inventory (LCI) involved the compilation and calculation procedures to determine all of the inputs and outputs of the product systems.^6^ In the present study, primary inventory data for the production of biogas and SCP were obtained from laboratory experiments. It was considered that, despite being direct data, they were not entirely representative, given that the laboratory used systems and technologies different from those that would be used on an industrial scale. Table 2 presents the inventory data at both laboratory and industrial scales.

**Table 2:** Life Cycle Inventory per 1 kg of Protein SCP at Industrial Scale and Lab Scale

		**Value – Industrial scale**	**Value – Lab scale**	**Unit**
*Feedstock preparation*				
Input	Electricity	1.65 × 10^–1^	6.64	kWh
Output	Homogenized waste	3.16 × 10^–1^	1.45 × 10^–1^	ton
*Anaerobic digestion*				
Input	Heat	1031	33.0	MJ
	Electricity	37.5	1.21	kWh
Output	Digestate	3.10 × 10^–1^	1.38 × 10^–1^	m^3^
	Biogas	7.20	7.06	m^3^
*Gas storage*				
Input	Electricity	7.91 × 10^–2^	0.00	kWh
Output	Biogas	7.20	7.06	m^3^
*Purification*				
Input	Activated carbon	2.00 × 10^–2^	8.50 × 10^–2^	kg
	Electricity	2.38	2.33	kWh
Output	Biogenic CH_4_	4.511	4.52	m^3^
	Biogenic CO_2_	2.69	2.54	m^3^
	Activated carbon	2.00 × 10^–2^	8.50 × 10^–2^	kg
*Bioreactor CSTR*				
Input	Electricity	8.48	126	kWh
	Medium (water and nutrients)	7.70 × 10^–1^	7.70 × 10^–1^	m^3^
Output	Biomass and unreacted medium	776	776	kg
	Emissions (biogenic CH_4_)	6.53 × 10^–1^	6.50 × 10^–1^	kg
*Separation – centrifugation*				
Input	Electricity	4.68	1.55	kWh
Output	SCP Biomass	75.4	73.4	kg
	Wastewater	7.01 × 10^–1^	7.03 × 10^–1^	m^3^
*Drying – spray dryer*				
Input	Electricity	3.69 × 10^–1^	3.69 × 10^–1^	kWh
Output	Biomass SCP (70.4% protein content)	1.42	1.43	kg
	Protein SCP	1	1	
	Emissions (water)	7.40 × 10^–2^	7.20 × 10^–2^	m^3^
	Emissions (particulates)	1.40 × 10^–2^	1.40 × 10^–1^	kg

In cases where the use of direct data was not possible—such as for chemicals, energy (both consumed and produced), fuels, avoided impacts of materials (i.e., fertilizers, energy, and biofuels), or waste management processes—data from the LCA database Ecoinvent 3.9 were used.^27^ The average electricity mix was used instead of the long-term marginal mix, as it was assumed that the increase in microbial protein production in Spain would have a negligible influence on the country’s total electricity demand.^28^

### Industrial Scale

To begin the ex-ante LCA, the first step involved developing a production scenario at an industrial scale and scaling up material flows and energy requirements.^29^ As shown in Figure 2, the industrial process for SCP production included six main units: (i) biowaste treatment, (ii) anaerobic digestion, (iii) biogas upgrading, (iv) SCP production, (v) SCP separation and drying, and (vi) digestate treatment.

Fish industry byproducts, including sludge, process wastewater, and product remains or discards, were used for subsequent SCP production. Considering available waste streams at a real-world scale and based on data provided by producing companies, the plant’s production capacity was set with an optimal annual flow of 1,800 tons of wastewater, 900 tons of solid waste, and 900 tons of sludge. From this annual amount, the daily substrate flow was determined, assuming it remains constant throughout the year. The parameters of the substrate composition are shown in Table S3. Energy consumption of the first unit were calculated according to the technical parameters of a wastewater feed pump and a commercial mixing pump (view Table S2).

In the AD unit process, a digester equipped with mechanical stirring and a digestate evacuation system was selected. To appropriately size this equipment, the required digester volume and hydraulic retention time (HRT) were calculated based on the characteristics of the input stream and the organic loading rate determined in the laboratory. As a result, the HRT was estimated at 40 days, and the organic loading rate, calculated based on volatile solids (VS), was 3.40 kg VS m^–3^ day^–1^. Under these conditions, the methane production efficiency was approximately 106.06 mL CH_2_ g^–1^VS. Additional parameters related to the AD unit are presented in Table S3. The energy demand for biomass mixing and pumping and the heat demand for AD were calculated based on the technical specifications of the equipment and the operating time, which depends on the process flow and the maximum capacity of the selected equipment. All parameters used for the energy flow calculations of this unit are presented in Tables S3 and S4.

The AD step generates two main output streams: biogas and digestate. The digestate is directed to a dewatering section. The liquid fraction was assumed to be treated in the existing wastewater treatment plant, while the solid fraction is sent to a composting unit. This solid digestate was characterized for its nitrogen and phosphorus content, using the molar mass of the corresponding avoided products—calcium nitrate and triple superphosphate, respectively—as reference values. The other output stream, biogas, consisted of approximately 64% CH_2_ and 36% CO_2_ by volume, with trace amounts of hydrogen sulfide (H_2_S).

In contrast to the lab scale, where the gas was stored in a bag, the biogas produced in the AD unit is assumed to be stored in a large gas holder and subsequently fed into a membrane-based cleaning system for H_2_S retention using activated carbon. The resulting product, primarily composed of CH_2_ and CO_2_, undergoes an additional purification process to obtain bio-CH_2_. In this step, approximately 1% of the biogas was lost and treated as air emissions. The SM provides detailed information on the parameters of the gas storage and purification unit, such as the H_2_S absorption coefficient in activated carbon and the biomethane retention coefficient. These parameters were used to calculate resource requirements, including the amount of activated carbon and the electricity consumption of the equipment.

Biomethane obtained from the previous process is introduced into the SCP bioreactor along with air. The bacteria are suspended in a liquid substrate composed of water with a specific proportion of nutrients that ensures optimal production. The reactor includes a pumping system for nutrient and gas input, which also functions as a mixing system, as well as a system for extracting the process liquid. The introduction of reaction medium in the specified amounts and proportions aims to ensure their bioavailability and the safety of the facility, based on the lab-scale design. The appropriate reactor volume and stirring equipment were selected based on the retention time and input flow rate. The required amount of nutrients was calculated by linearly scaling up the laboratory data,^30^ while energy consumption was determined based on the operating time and power specifications of commercial equipment, as detailed in the SM.

A centrifugation system was used to concentrate the SCP biomass to 98.1%. And, in a spray-drying unit process, moisture was removed from the SCP biomass, assuming a 99.0% moisture reduction,^10^ resulting in dry SCP biomass in particle form with a protein content of approximately 70.0%. The inventory data for these two units was obtained from Vea et al.^31^ The effluents generated during the centrifugation of SCP biomass, as well as those derived from the treatment of digestate produced in the AD unit, were assumed to be treated using a conventional wastewater treatment process based on data from the Ecoinvent 3.9 database.

After the scale-up of each unit process was completed, all steps were connected, including primarily the pumping requirements between equipment. Additionally, it was assumed that the reactors were optimally insulated, and heat losses were considered minimal, following a conservative approach.^29^

### Avoided Impacts: Energy and Materials

Recovered materials or energy produced as byproducts (i.e., compost, electricity and/or heat), were accounted for by expanding the system boundaries to include avoided primary productions, thereby mitigating the environmental burdens associated with their production.^32^

The compost obtained from the solid fraction of the digestate, after being treated with a composting system, was assumed to replace commercial inorganic fertilizers,^33^ in accordance with current regulations on fertilizer products.^34^ For replacing inorganic fertilizers, the nutrient distribution within the compost was retrieved from the literature: N (as TKN), K (as K_2_O), and P (as P_2_O_5_) were assumed to be equal to 6.8, 4.2, and 10.2 g/kg_TotalSolids_ of compost, respectively.^35^ The procedure followed to calculate the substitution ratio of each inorganic fertilizer is illustrated in the SI. In this case, average production data for calcium ammonium nitrate and triple-super phosphate was used as a proxy for lacking marginal data on fertilizer production.^36^

Regarding the biomethane that is not used for SCP production, it was assumed in the baseline scenario that it was directly emitted into the atmosphere. In the other scenarios, the valorization of this surplus was incorporated. In Sc1, the surplus biomethane recovered from the biogas upgrading process was assumed to avoid the production and combustion of natural gas, considering a purity of biomethane equal to 97.92% and an electricity consumption of 0.07 kWh/kg CH_4_ for the compression and injection of biomethane into the grid.^37^ The foreseeable consequence of the use of biogas is, in a short-term perspective, that marginally produced natural gas will be replaced. While in Sc2, the surplus biomethane fed a CHP plant, assuming an efficiency of 36% for electricity generation and 60% for heat generation.^38^ The electricity produced displaces medium-voltage electricity from the Spanish grid, while the thermal energy produced displaces heat generated by natural gas boilers. Finally, in scenario 3, the energy produced in the CHP plant was used to meet the thermal and electrical needs of the internal processes, thus reducing external energy consumption. The appropriate Ecoinvent records for Spanish electric and thermal energy were used (Table S3).

### Life Cycle Impact Assessment

To translate the inventory data into environmental impacts, the SimaPro v9.6 software was used. The selection of midpoint assessment indicators was guided by the recommendations outlined in the Product Environmental Footprint Category Rules (PEFCR),^39^ which are included in the Environmental Footprint 3.1 (EF 3.1) method.^40^ The impact categories studied were selected based on their relevance to the LCA of microbial protein production.^6^ Specifically, this assessment considers the following impact categories (acronyms and units in brackets): acidification potential (AP, mole of H^+^ eq), climate change (CC, kg CO_2_ eq), freshwater eutrophication (FE, kg P eq), marine eutrophication (ME, kg N eq), land use (LU, Pt), and water use (WU, m^3^ depriv.).

Additionally, a Monte Carlo (MC) simulation was conducted since it is the most adequate approach to integrate uncertainty in LCA. This involves performing multiple simulations, where each one utilizes random values for the input variables that exhibit uncertainty, allowing for the calculation of impact assessment results while considering the variability in inventory flows. For the current study, pseudorandom values (i.e., 500 iterations) were generated for each data point, following their probability distribution based on the Pedigree matrix.^41^ This analysis is provided in the Supporting Information.

### Sensitivity and Uncertainty Analysis

A sensitivity analysis was conducted to assess the uncertainty of the parameters and scenarios selected in this study. As aforementioned, the system expansion method was chosen to address the coproduction inherent in the management of byproducts from the fishing industry, which were used to obtain SCP. However, one of the main challenges in LCA is dealing with multifunctionality, where a process produces multiple products. ISO 14040 recommends avoiding allocation, but in cases where multifunctionality is unavoidable, it suggests the use of system expansion.^26^ Otherwise, practitioners should consider another allocation method based on physical, or based on the economic value of the different functions, or use another method to account for multifunctionality in LCA. To ascertain the extent to which the choice of allocation method affects the environmental impact results, a sensitivity analysis was performed in which physical allocation and economic allocation were considered as alternatives to system expansion. The procedures for physical (or mass) allocation and economic allocation involve partitioning the total environmental burden of a process that produces multiple products, proportionally to the individual production of each product. The allocation coefficients used for each product, along with the specific calculation procedures, can be found in the SI.

On the other hand, electricity supply is known to be a significant contributor to the total impact of SCP production.^42^ Therefore, the influence of different Spanish electricity sources was analyzed. This included the Spanish grid mix, as well as electricity with guarantees of origin (GOs) and the residual mix. The former allows consumers to trace the origin of the electricity, ensuring that it comes from renewable sources or high-efficiency cogeneration.^43^ However, not all electricity generated can be traced through GO certificates, leading to the definition of a residual mix that includes the portion of electricity generation not covered by traceable renewable sources. Information regarding the share of each energy source in the different electricity systems was retrieved from the report provided by AIB^25^ and is presented in the Supporting Information.

## Results and Discussion

### Life Cycle Impact Assessment and Scenario Analysis

Table 3 presents the results of the life cycle impact assessment bases on the FU of 1 kg of dry protein. It is important to note that positive indicators represent adverse environmental impacts, whereas negative indicators denote environmental benefits. To determine the net impacts or savings, avoided impacts were subtracted from induced impacts. As previously discussed, the baseline scenario assumed no changes in the future commercial-scale production process compared to the current lab scale. This scenario was used as the baseline for comparisons throughout the discussion.

**Table 3:**
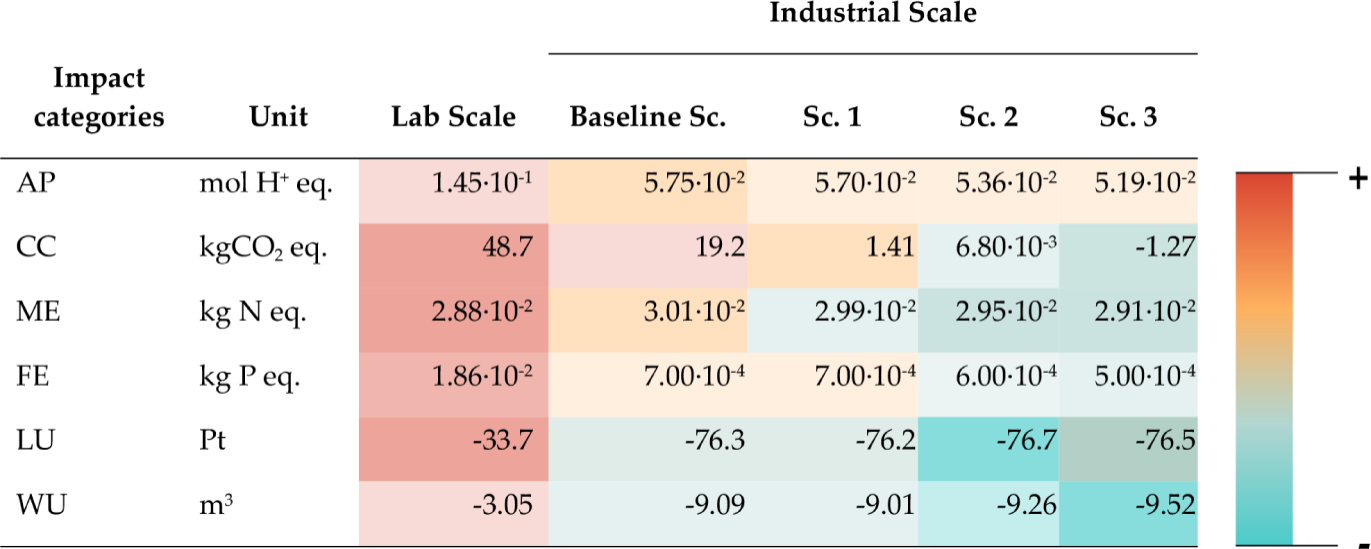
Life Cycle Impact Assessment Results for Each Scenario per FU Defined as 1 kg of Dry SCP

As shown in Table 3, the lab-scale process exhibits the highest environmental impacts, with reductions ranging from 60% to 96% across all analyzed impact categories when scaled up. The largest differences were observed in eutrophication (marine and freshwater), followed by water consumption, land use, and acidification. Specifically, for the climate change impact category, the impact decreased by a factor of 2.5 in the baseline scenario. These findings align with those of Röder and colleagues,^30^ who evaluated a novel production process for proteins derived from rapeseed cake. The substantial differences between lab and industrial scales can be attributed primarily to two factors. First, the implementation of industrial-scale equipment and process design significantly reduces potential environmental impacts.^29^ Second, improvements in process efficiency, reuse and recycling of raw materials, use of residual heat, and continuous processes instead of batch processes that consume higher energy levels, are only feasible beyond a certain production volume.^30^ Therefore, it can be concluded that LCAs based on laboratory data may overestimate environmental impacts, as optimal yields and efficiencies are not achieved until a certain production scale is reached.^44^ This underscores the importance of scaling when conducting a prospective assessment, particularly for evaluations focused on emerging technologies or processes at early stages of development, which consequently have a low technology readiness level (TRL).

At industrial scale, the baseline scenario demonstrated the poorest environmental performance across all impact categories, primarily due to biogenic methane emissions resulting from the proportion of CH_2_ that was not utilized for methane production, as illustrated in the contribution analysis. In contrast, the modified scenarios exhibited the lowest environmental impacts, as they incorporated various valorization pathways for biomethane, thus reducing biogenic emission levels to only fugitive emissions during the gas storage and transport stages.

For the CC category, the baseline scenario, 19.24 kg CO_2_ eq, showed environmental impacts significantly higher than those previously reported by Aidoo et al.^7^ for SCP production from crude pea starch, estimated at 0.61 kg CO_2_ eq per kg of protein. Similarly, the baseline scenario also surpassed the environmental loads obtained by Järviö et al.,^45^ who considered a production system with hydrogen-oxidizing bacteria powered by hydropower, achieving 1.6 kg CO_2_ eq per kg of protein. However, other authors reported considerably higher values for protein production using rice straw as a substrate^18^ and yeast-based microbial protein derived from oat residues,^12^ with approximately 23 and 22 kg CO_2_ eq per kg of protein, respectively. The comparison of results is complex, as each author adopts different methodological approaches and assumptions. Discrepancies between the results can be attributed to variations in system configurations, the technologies employed, or the materials used in each process. Therefore, it is crucial to account for these variables when interpreting the results and assessing the environmental and technological feasibility of the proposed alternatives.^6^

The modified scenarios achieved better environmental performance than the baseline across all impact categories. Scenario 1, in which the biomethane remaining from SCP production is assumed equivalent to substituting natural gas as a commercial product, showed environmental improvements over the baseline in categories such as CC (−93%) and AP (−1%). Meanwhile, Scenarios 2 and 3, in which 22% of the biogas is upgraded to biomethane and assumed to cogenerate heat and electricity, demonstrated the best environmental performance among those investigating bioCH_2_ recovery, with notable reductions in CC impact (−99% and −107%), AP (−6.8% and −9.8%), and eutrophication categories (−3.4% and −23%), among others. The difference between the scenarios at industrial scale was small except for CC and AP.

Overall, Scenarios 2 and 3 achieved the best results. From an environmental perspective, the findings in this study suggest that the optimal configuration may lie in the combined recovery of biomethane, electricity, and heat, balancing the biomethane allocation between CHP and SCP production. This result highlights an opportunity for further investigation into the applicability of this modified baseline scenario in an industrial facility.

### Contribution Analysis

This section presents the contributions of the system’s key processes and subprocesses. Figure 3 distinguishes two types of loads: avoided impacts, shown below the axis (e.g., avoided electricity and heat production, grid gas production, and inorganic fertilizer production), and induced impacts, shown above the axis (e.g., substrate pretreatments, AD, biogas storage and purification, biomass reactor, biomass separation, SCP drying, composting, and energy recovery devices). The net impact for each scenario is calculated by subtracting avoided impacts from induced impacts. A negative net balance represents “savings,” while a positive net balance indicates an “impact.” Additionally, SM reported the contributions of individual flows to each subprocess.

**Figure 3:**
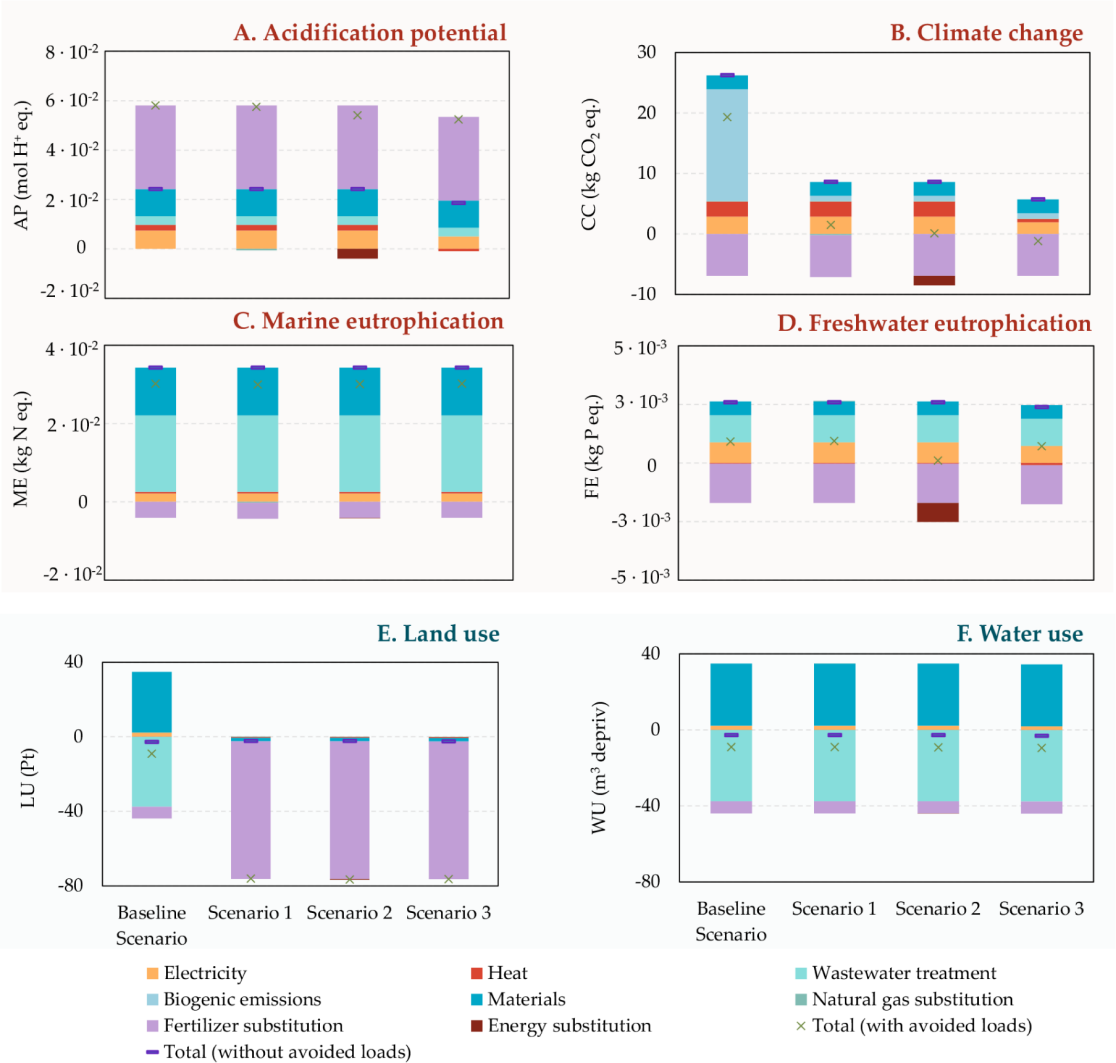
Contribution analysis of SCP processing at industrial scale, considering avoided loads to (A) acidification potential, (B) climate change, (C) marine eutrophication, (D) freshwater eutrophication, (E) land use, and (F) water use.

Regarding acidification, the primary contributions to avoided impacts come from biofertilizer production, with a smaller contribution from biomethane production. Across all analyzed scenarios, the avoided emissions associated with biofertilizer were −0.07 kg SO_2_ eq per kg of SCP produced, representing a 38% reduction. In contrast, biomethane recovery had a less pronounced effect on acidification potential. In terms of SO_2_-equivalent emissions, the environmental benefits from natural gas production contributed 1% in scenario 2, while electricity and heat cogeneration accounted for a 6.37% reduction in scenario 3. It is important to highlight that the displacement of inorganic fertilizer production played a critical role in reducing acidification impacts.

Regarding direct impacts, primary contributors to AP were SO_2_-equivalent emissions associated with digestate treatment for biofertilizer production, accounting for approximately 50% of the total in all scenarios. Therefore, when adding the avoided burdens from the substitution of conventional fertilizers (as previously discussed) to the environmental burdens of biofertilizer production, the net AP impact remains positive, as shown in Figure 3A. Contributions from electricity and heat consumption, from nutrient inclusion, and from supernatant treatment were also significant across all scenarios investigated (see Figure 3A).

For the CC impact category, the avoided production of inorganic fertilizers accounted for a substantial portion of the avoided impacts, contributing 21% in the base scenario, 44% in Scenario 1, 40% in Scenario 2, and 55% in Scenario 3 (see Figure 3B). Biomethane production also contributed to avoided emissions, ranging from 2% to 10% in Scenarios 2 and 3, respectively. In all modified scenarios, the avoided impacts were greater than the direct impacts (positive burdens), demonstrating that the valorization of residual biomethane from SCP production for combined heat and power cogeneration or as natural gas is essential to mitigate the CC impact.

In the baseline scenario, a fraction of the biomethane introduced into the reactor is released back into the atmosphere, contributing 55.63% to the total impact in the CC category. In contrast, in the modified scenarios, this biomethane is valorized as an energy source. As a result, the baseline scenario showed the highest impact. Additionally, the AD stages and the reactor were the main contributors to the system’s negative impacts, primarily due to the high energy requirements. Consequently, the contribution of electricity and thermal energy consumption was notable across the different scenarios, representing between 17% and 34% of the direct impacts. In the scenario that included CHP generation in the later phase (Sc.3), heat and electricity were supplied internally, so no additional environmental load was assumed for the process. Under these circumstances, emissions resulting from biogas combustion in the CHP plant were allocated solely to the cogeneration unit. In contrast, in the other scenarios, heat and electricity were sourced externally, introducing an additional environmental burden from the background system. This explains the better environmental performance of Sc.3, where the avoided burdens outweighed the environmental loads, compared to the other scenarios.

The eutrophication potential, both marine and freshwater, was dominated by waste disposal and supernatant treatment, which accounted for 26% to 55% of the positive impacts, as shown in Figures 3C,D. Subsequently, the nutrients used in the SCP cultivation reactor significantly contributed to this category, with contribution percentages of approximately 30% for ME and 14% for FE. However, the avoided production of inorganic fertilizers generated substantial environmental credits, as well as the avoided production of heat and electricity in Sc.2.

Finally, regarding resource use categories, it is noteworthy that both land use and water use categories show an environmental benefit, as the avoided burdens exceed the direct impacts. Specifically, environmental credits are assigned for the avoided burdens of producing conventional fertilizers, leading to negative impacts in land use (−37%) and water use (−8%). Synthetic fertilizer production often involves the extraction of raw materials, such as phosphate and potassium, which requires large land areas. Additionally, fertilizer production consumes significant amounts of water, both in resource extraction and in industrial synthesis processes, especially for ammonia and urea production. The treatment of the wastewater from the analyzed process reduces the demand for new water sources, leading to a decrease in environmental burdens in the water use category (−48%).

### Sensitivity Analysis

As shown earlier, the electricity consumption footprint was unavoidable across all scenarios, whether as an induced impact or as an energy substitution. For this study, the baseline technology was assumed to be a mixed energy source, comprising renewable energy sources (50.55%), coal-based electricity (28.21%), and nuclear energy (20.95%), based on the AIB report for the year 2023.^25^ A sensitivity analysis was conducted to examine how the environmental impacts would change if either fully renewable electricity or residual electricity were selected as the marginal technology. As expected, the use of electricity with GO yielded the best environmental performance due to the reliance on renewable sources, while the use of the residual mix exhibited the worst environmental performance. As shown in Figure 4, the modified scenarios exhibited the greatest sensitivity to these changes. This sensitivity analysis focuses exclusively on greenhouse gas (GHG) emissions due to their global urgency and the robust availability of data in this area. GHG emissions decreased by 9% compared to the use of regular electricity in the baseline scenario and by approximately 24% in the three modified scenarios. In contrast, the use of residual electricity resulted in a 10% increase in the baseline scenario and a little over 25% in the modified scenarios. It is important to note that these effects may have been underestimated, as the use of renewable energy as an input was not considered in the production processes of raw materials in this sensitivity analysis.

**Figure 4:**
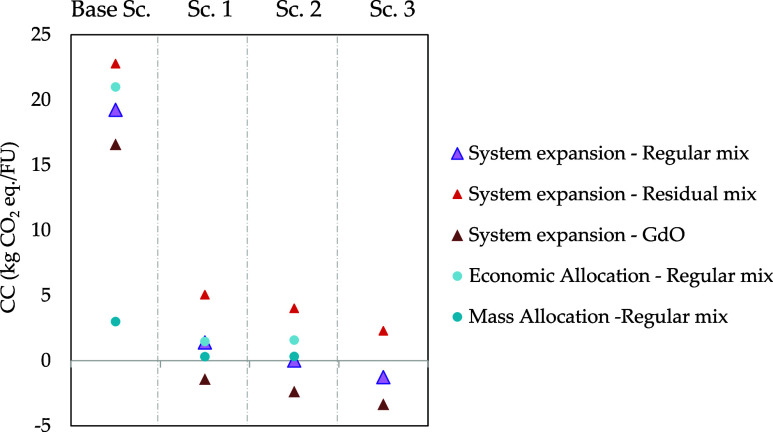
Sensitivity analysis results for the different scenarios, considering different electricity sources and different allocation methods.

On the other hand, allocation methods constitute one of the major sources of uncertainty in LCA results. That is why, in this sensitivity analysis, allocation based on mass and based on economic value were applied instead of system expansion for dividing burden between coproducts. In allocation methods, all inputs and outputs of the production system are attributed to the functional unit by linking and/or partitioning the unit processes within the system according to a normative rule (i.e., allocation factors). Figure 4 illustrates how environmental impacts in terms of CC significantly decrease when mass-based allocation is used, with SCP protein as the main product. In the baseline scenario, a reduction of 78% was achieved when applying mass allocation. This is attributed to the fact that the production level of protein is ten times lower than that of biofertilizer, leading to an underestimation of impacts per FU. In contrast, when economic allocation is employed, environmental impacts increase slightly (by 5%), as the value of the coproducts is significantly lower than that of the main product. Although the price of SCP protein remains uncertain due to its current absence in the market, this economic allocation reflects the disparity in value.

The choice of economic allocation generally favors coproducts, as the main product typically holds a higher economic value, and it exhibits high sensitivity to market volatility. In contrast, mass allocation provides greater temporal stability than economic allocation, as products that are functionally more valuable receive less impact allocation if they have a smaller mass. Therefore, it can be concluded that system expansion presents a solution to avoid complex issues related to allocation, constituting an effective method for assessing environmental impacts in multifunctionality contexts. When environmental burdens and impacts are distributed among different coproducts, each of them carries a smaller burden, thus leading to the conclusion that the process generates global environmental benefits due to the “avoided burdens” arising from the valorization of waste and byproducts.

### Challenges in Implementing Ex-Ante LCA

Throughout this study, it has been demonstrated that LCA is a powerful tool for evaluating emerging technologies, providing valuable information to guide the early development of these technologies. However, the traditional application of LCA, which primarily focuses on current and short-term technologies, is not always suitable for technologies under development that are not yet fully established. This creates a need to adapt LCA to address the specificities and uncertainties inherent in emerging technologies, giving rise to the concept of ex-ante LCA.^46^

LCA results are subject to uncertainties arising from both methodological approaches and the assumptions made throughout the study. These issues are amplified when applied to technologies with low TRLs. Various studies, such as those conducted by Cucurachi et al.^47^ and Moni et al.,^44^ have highlighted the challenges inherent in future-oriented LCA.

One of the main obstacles in a future oriented LCA is the lack of reliable data on future processes. Since this type of analysis is based on technologies and systems that have not yet been implemented or commercially scaled, the availability of real data is very limited. While estimates and projections can be used, these will always be subject to a high degree of uncertainty. In this study, laboratory-scale data was available, and the industrial-scale process was derived by scaling the data based on production capacity and industrial equipment. However, scaling itself presents additional challenges. While production stages are similar at both scales, the industrial scale can introduce significant changes in material flows, energy efficiency, and resource use.^44^ To carry out this, it is essential to develop practical guidelines, such as those by Piccino and collaborators which integrate process design and scaling with LCA studies.^29^ In this study, the scaling process was simplified through estimates based on bibliographic references and validated through scenarios supported by expert partners, who provided specialized knowledge.

Another important challenge in developing the ex-ante LCA was avoiding a temporal mismatch between foreground and background systems, as the latter can change over time.^46^ Current background systems may be relevant if it is believed they will remain constant over an extended period. However, the further into the saturation phase the technology is expected to go, the more critical it becomes to consider developments in background systems. To address time-related changes, marginal supplies were used, as they allow the LCA model to capture dynamic changes in background systems as emerging technologies are scaled, and the demand for resources shifts. However, market demand and consumption patterns were not considered in this study.

Finally, since uncertainty is an inherent characteristic of any future-oriented study, such as prospective LCA, it must be managed and communicated clearly and transparently. The results obtained from a ex-ante LCA should, therefore, be interpreted with caution, as environmental impact projections are sensitive to the assumptions and scenarios used. However, these results are valuable as they provide insights into potential consequences and allow decision-makers to proactively identify critical points, optimizing processes to avoid potential impacts. Moreover, due to the limited literature focused on ex-ante LCA for industrial processes, comparing and verifying the reliability of the results with other studies is difficult.

While this study provides valuable insights into ex-ante LCA modeling and microbial protein production systems, some limitations must be noted. First, the study relies on various assumptions and simplifications inherent to the modeling framework; thus, the findings should be interpreted as indicators of potential future impacts, rather than absolute predictions. Additionally, the accuracy of the results largely depends on the quality and availability of input data, which may vary between regions and databases. Finally, as a recommendation for future research, the analysis does not account for potential changes in technologies and policies, which could significantly influence the results.

## Conclusion

This study demonstrated the implementation of LCA methodology and assessed the scaling-up process for a novel production pathway, focusing on the future-oriented evaluation of single-cell protein from biomethane derived from the AD of byproducts from the food processing industry. The findings reveal that these byproducts have a high potential for biorefining into value-added products. Consequently, different novel valorization pathways were explored, including microbial protein, biomethane, and bioenergy.

Based on laboratory-scale experiments, various biorefinery scenarios were developed to investigate bioresidue-based alternatives for producing bioenergy and microbial protein, and their sustainability was investigated from an environmental perspective. The alternatives that studied the production of both SCP protein and bioenergy (i.e., combined heat and power cogeneration) resulted in net environmental savings in most of the environmental indicators assessed. The findings demonstrated that the environmental benefits of each scenario depend not only on the biorefining pathway but also on the downstream processing strategies. For instance, in the climate change damage category, cogeneration in a CHP plant generated greater savings than using biomethane as grid gas, i.e., 0.007 versus −1.270 kg CO_2_ eq/kg of SCP protein, respectively. In this context, microbial protein production with upgraded biogas and recirculation of the bioenergy generated from the cogeneration plant for internal use (Sc3) emerged as the most environmentally favorable pathway for biowaste valorization. The Sc3 scenario showed potential for reducing environmental impacts by 10% to 110% across all evaluated categories compared to the baseline scenario.

The comparison between laboratory-scale and industrial-scale outcomes highlighted the significant influence of scalability on LCA results. This study underscores the challenges inherent in ex-ante LCA, such as the lack of data and established procedures for scaling up, as well as the inherent uncertainty in future projections. Nonetheless, ex-ante LCA proved its potential to assess the environmental impact of early stage processes while considering industrial scaling, identifying critical environmental hotspots, optimizing designs prior to scaling, and generating valuable data for strategic decision-making. Thus, despite inherent uncertainties, ex-ante LCA provides valuable insights for decision-makers and developers of emerging technologies.

## Supplementary Material



## Notes

Process modeling details; avoid production of energy and material; sensitivity analysis, allocation; sensitivity analysis, electricity mixes; uncertainty analysis (PDF)
