# Supplementary material for: Environmental Insights into Single-Cell Protein Production: A Life Cycle Assessment Framework
Source: ACS Sustain Chem Eng. 2025 May 31;13(23):8699–710. doi: 10.1021/acssuschemeng.5c02336 (PMC12244465; doi:10.1021/acssuschemeng.5c02336)
Supplement: Supplementary file 1 [file sc5c02336_si_001.pdf]

Supporting Information

Environmental insights into single cell protein production – A life cycle assessment framework

Eva Martínez-Ibáñez[a,*], Jara Laso[a], Marta María Pérez-Martínez[b], Raquel Martínez-Vazquez[b], David Baptista de Sousa[c], Diego Méndez[c], Elena Olaya-Pérez[b], Virginia Marchisio[b], Ruben Aldaco[a], María Margallo[a]

[a] Department of Chemical and Biomolecular Engineering, University of Cantabria, Av. Los Castros s/n, Santander, Cantabria, 39005, Spain
[b] EnergyLab Technology Center, Fonte das Abelleiras s/n, Vigo, Galicia, 36310, Spain
[c] ANFACO-CECOPESCA, Department of Sustainability and Circular Economy, Campus University 16, Vigo, Galicia, 36310, Spain

*Corresponding author: martinezie@unican.es

Total number of pages: 9 (S1-S19)

Total number of figures: 1 (Figure S1)

Total number of tables: 13 (Table S1-Table S13)

Section I. Process modeling

This section outlines the parameters and values used to model anaerobic digestion and the SCP reactor. The numbers presented below represent base case values at an industrial scale. The product system's relevant energy forms include heating for reactors, as well as energy for stirring, filtration, pumping, and drying. Energy input calculations were manually performed, considering operational flow rates and the power requirements of each unit.

Table S1: Substrate composition parameters.

Parameter	Unit	Value
Flow	m^3/d	9.86
Dry weight (total solids)	wt%	14.50
Total volatile solids	kg VS / d	1,356
Volatile Solids Percent	dry wt%	94.83

Table S2: Pretreatment parameters.

Parameter	Unit	Value	Reference
Substrate flow	m^3/d	9.86	Current study
Water flow	m^3/d	4.93	Current study
Power (Mixing Pump)	kW	7.5	Commercial Pump
Power (Water Pumping)	kW	15	

Table S3: Anaerobic digester parameters.

Parameter	Unit	Value	Reference
Hydraulic Retention Rate	Days	40	Current study
Digester volume	m^3	395	Current study
Organic Loading Rate	kg VS/m^3/d	3.40	Current study
Methane Yield	mL/g VS	106.06	Current study
Volatile Solids Percent	%	94.83	Current study
Biogas Methane Percent	%	63.94	Current study
Biogas Carbon Dioxide Percent	%	36.06	Current study
Biogas impurities (e.g., H_2S)	ppm	567	Current study

$$\text{Organic Loading Rate}\left(\frac{\text{kg VS}}{\text{m}^3\text{ d}}\right) = \text{total VS}\left(\frac{\text{kg VS}}{\text{d}}\right) * \text{Digester volume (m}^3) \quad \text{(S1)}$$

$$Dige\text{ster volume (m}^3) = \text{Flow}\left(\frac{\text{m}^3}{\text{d}}\right) * \text{Hydraulic Retention Rate (d)} \quad \text{(S2)}$$

Table S4: Anaerobic digestion and biogas storage parameters.

Parameter	Unit	Value	Reference
Yield (biogas)	m^3 biogas / ton substrate	22.81	Current study
Substrate flow	m^3/d	9.86	
Recirculation pump	kW	7.5	Commercial equipment
Power (agitation)	kW	11	
Gasometer capacity	m^3	100	
Power (compression)	kW	1.5	

Table S5: Membrane filtration (biogas purification) parameters.

Parameter	Unit	Value	Reference
Yield (bioCH_4)	m^3 bioCH_4 / ton substrate	14.3	Current study
Biomethane Retention	%	100	Current study
Absorption Coefficient	kgH_2S/kg activated carbon	0.35	Current study
Activated Carbon Lifespan	Days	1.7	Current study
Power Consumption	kWh/m^3 biogas	0.33	Current study

Table S6: Bioreactor (CSTR reactor) parameters.

Parameter	Unit	Value	Reference
Yield (raw SCP)	kg SCP_{raw}/kg substrate	0.24	
Hydraulic Retention Time	Days	2	
Biomethane flow	m^3/day	140.96	
Air flow	m^3/day	1751.2	
Water + nutrients flow	m^3/day	24.19	
Power (nutrients pumping)	kW	0.75	Commercial equipment
Power (aeration)	kW	15	

Table S7: Centrifugation (biomass separation) parameters.

Parameter	Unit	Value	Reference
Biomass Retention	%	98.10	Current study
Power Consumption	kWh/kg biomass	0.06	(1)

Table S8: Spray Drying parameters.

Parameter	Unit	Value	Reference
Biomass Retention	%	99.00	(2)
Power Consumption	kWh/kg biomass	0.26	(1)

Section II. Avoided production of energy and material

Natural gas, electricity, heat, and materials recovered from biowaste treatment were considered as avoided commercial products, thereby mitigating the environmental burdens associated with their production. The avoided impacts were accounted incorporating Ecoinvent processes into the modeling that adequately characterize these products.[3]

The solid fraction of the digestate obtained through centrifugation of the digestate was subjected to a composting process. The inventory of this process was obtained from the literature[4] and is shown in Table S9.

Table S9: Summarized inventory data for the digestate compost treatment. Data reported per FU: 1 kg of compost.

Input/output	Unit	Value
Input		
Electricity	kwh	0.0665
Heat	kwh	0.0623
Sulfuric acid	kg	0.00400
Digestate	kg	1.25
Output-Emissions		
CH4	kg	1.17E-03
N20	kg	1.21E-04
NH3	kg	8.81E-04
NOX	kg	8.98E-06
NO3-	kg	8.84E-04
Output-Product		
Compost	kg	1

It was assumed that the compost obtained, considering the nutrient content (Table S10), replaces the commercial inorganic fertilizer NPK15. Assuming that potassium and phosphorus have a 100% uptake ratio, suggesting that they are as affective as mineral fertilizers, while the nitrogen substitution percentage is assumed to be approximately 40% due to its lower bioavailability.[5] It was found that the substitution rate was 1 kg of compost for 0.2 kg of inorganic fertilizer.

Table S10: Nutrient content of the compost produced from composting dry digestate.

Parameter	Unit	Value	Reference
Dry matter	%	25.5	(4)
Nitrogen (as TKN)	g/kg_{TS}	6.8	(4)
Phosphorous (as P_2O_5)	g/kg_{TS}	10.2	(4)
Potassium (as K_2O)	g/kg_{TS}	4.2	(4)

Regarding the biomethane that is not used for SCP production, it was assumed in the baseline scenario that it was directly emitted into the atmosphere. In the other scenarios, the valorization of this surplus was incorporated. In Sc1, the surplus biomethane recovered from the biogas upgrading process was assumed to avoid the production and combustion of natural gas, considering a purity of biomethane equal to 99.92% and an electricity consumption of 0.07 kWh/kgCH_4 for the compression and injection of biomethane into the grid.[6] While in Sc2, the surplus biomethane fed a cogeneration heat and power (CHP) plant, assuming an efficiency of 36% for electricity generation and 60% for heat generation. The electricity produced displaces medium-voltage electricity from the Spanish grid, while the thermal energy produced displaces heat generated by natural gas boilers. Finally, in scenario 3, the energy produced in the CHP plant was used to meet the thermal and electrical needs of the internal processes, thus reducing external energy consumption. Table S11 presents the Ecoinvent processes used to account for the avoided impacts of biomethane recovery, thermal energy and electricity from the CHP unit, and inorganic fertilizers.

Table S11: Ecoinvent processes for avoided impacts of biogas upgrading and compost.

Avoided impact	Ecoinvent process
Thermal energy - CHP unit	Heat, central or small-scale, natural gas {Europe}ǀ heat production, natural gas, at boiler modulating <100kW ǀ Conseq, U
Electricity – CHP unit	Spanish electricity residual mix ǀ Conseq, U
Natural gas recovery from biomethane	Natural gas, high pressure {ES}ǀ market for ǀ Conseq, U

Inorganic fertilizers	Inorganic nitrogen fertiliser, as N {ES}| market for inorganic nitrogen fertiliser, as N | Conseq, U<br>Inorganic phosphorus fertiliser, as P2O5 {ES}| market for inorganic phosphorus fertiliser, as P2O5 | Conseq, U<br>Inorganic potassium fertiliser, as K2O {ES}| market for inorganic potassium fertiliser, as K2O | Conseq, U

Section III. Sensitivity analysis – Allocation

Table S12: Mass and economic allocation coefficient (%) of main product and co-products.

Product type	Mass allocation coefficient	Economic allocation coefficient
SCP (Main product)	2.49%	11.97%
Fertilizer (Co-product)	95.78%	83.90%
Natural gas (Co-product)	1.72%	4.13%

Section III. Sensitivity analysis – Electricity mixes

Table S13: Contribution (%) of each energy source to the different electricity mixe.[7] Note: RE (Renewable energy) and FO (Fossil origin)

Source	Regular mix (%)	Residual mix (%)	Guarantee of origin (%)
RE unspecified	0.29	0	0
RE biomass	1.44	0	4
RE solar	16.3	1.89	21.5
RE wind	24.18	1.51	50.62
RE hydro	8.63	0	23.16
Nuclear	20.95	37.51	0
FO unspecified	0	0.62	0
FO hard coal	1.5	8.17	0
FO lignite	0	0.02	0
FO oil	1.17	2.26	0
FO gas	25.54	48.03	0

Section IV. Uncertainty analysis

This section shows the results of the uncertainty analysis carried out for Climate Change (CC) indicator for SCP production following a Monte Carlo (MC) simulation.

This section shows the results of the sensitivity analysis carried out for all indicators for hydroponic tomato production following a MC simulation. The main objective of this type of simulation is to produce a random deviation considering the specified probability distribution of

parameters.[8] SimaPro LCA software support this method applied to propagate uncertainty in LCA. MC utilizes repeated random sampling to generate simulated data that is then used with a mathematical model, allowing the calculation of impact assessment results while accounting for variability in the inventory flows. The propagated uncertainty was obtained by generating a geometrically distributed uncertainty interval from the Monte Carlo-generated sample space. Within this interval, the 97.5th percentile represented the upper limit value, while the 2.5th percentile represented the lower limit. For the current study, pseudo-random values (i.e., 500 iterations) were generated for each data point, following their probability distribution based on their Pedigree matrix.

The Pedigree matrix is the mechanism used to convert a qualification of each input or output flow into a quantitative value, considering the following indicators: reliability, completeness and temporal, geographic and technological correlation.[9] It should be noted that ca. 75% of process units available in the ecoinvent® v3.4 dataset include a lognormal distribution calculated with the pedigree matrix, whereas most of the remaining datasets lack any sort of distribution and generated using geometric standard deviations, as the sample size of the inventoried processes was not sufficiently large to perform a regular normal distribution.

Figure S.1. depicts the probability density distribution histograms for the climate change category in the three scenarios considered, with a 95% of coverage probability and a total of 500 iterations. In all cases, GWP impacts followed a normal distribution with an average value of 19 kg, 1.31 kg, and 0.11 kg CO_2 eq annually, with a standard deviation of 1.5 kg, 1.5 kg, and 1.84 kg CO_2 eq. for the baseline, modified 1, and modified 2 scenarios, respectively. The highest coefficient of variation (CV) among the alternatives was estimated at 16.7% for the scenario 3, which represent the greater level of dispersion around the mean. It is worth noting that for all the input distributions of the modified scenarios, not even 2.5% of the randomly simulated combinations (line representing 97.5%) are above the average for the baseline scenario. In other words, based on the available data, it is virtually impossible that the alternative future scenarios emit more greenhouse gas emissions than the current one under any possible condition.

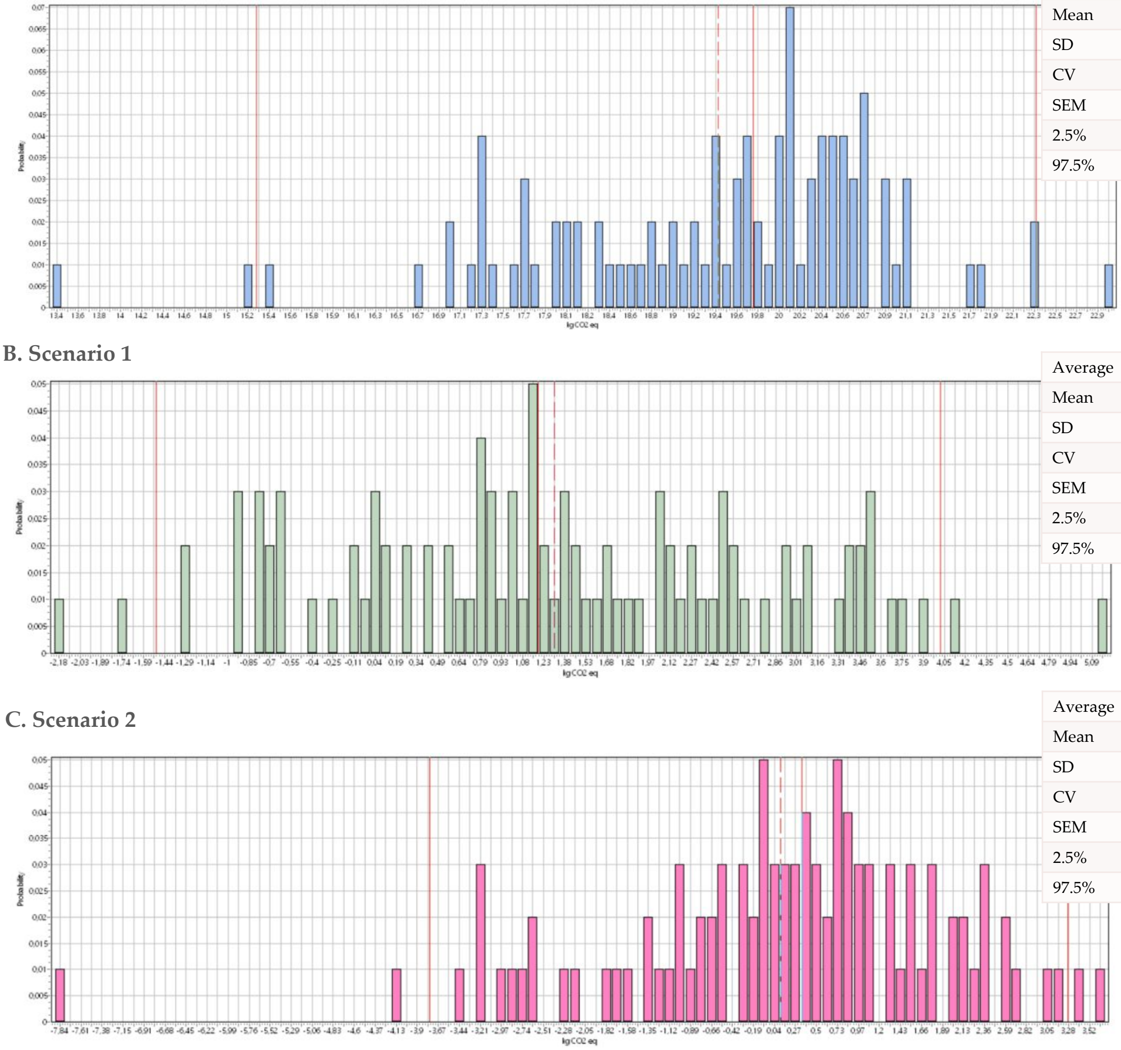

A. Baseline scenario

Average	19
Mean	19.3
SD	1.5
CV	8.03%
SEM	0.15
2.5%	15.2
97.5%	21.6

B. Scenario 1

Average	1.31
Mean	1.19
SD	1.5
CV	115%
SEM	0.15
2.5%	-1.5
97.5%	4.02

C. Scenario 2

Average	0.11
Mean	0.34
SD	1.84
CV	1670%
SEM	0.18
2.5%	-3.77
97.5%	3.28

Figure S1: Probability density distribution of CC in SCP production. (a) Baseline scenario, (b) Scenario 1, (c) Scenario 2. SD: standard deviation; CV: coefficient of variation; SEM: standard error of the mean.

References

(1). Vea, E.B., Fabbri, S., Spierling, S., Owsianiak, M., 2021. Inclusion of Multiple Climate Tipping as a New Impact Category in Life Cycle Assessment of Polyhydroxyalkanoate (PHA)-Based Plastics. Sci. Tot. Environ. 788. 147544. https://doi.org/10.1016/j.scitotenv.2021.147544

(2). LaTurner, Z.W., Bennet, G.N., San, K.Y., Stadler, L.B., 2020. Single Cell Protein Production From Food Waste Using Purple Nonsulfur Bacteria Shows Economically Viable Protein Products Have Higher Environmental Impacts. J. Clean. Prod. 276, 123114. https://doi.org/10.1016/j.jclepro.2020.123114

(3). Cucurachi, S., Steubing, B., Siebler, F., Navarre, N., Caldeira, C., Sala, S., 2022. Prospective LCA methodology for Novel and Emerging Technologies for BIO-based products - The PLANET BIO project. Luxembourg. JRC129632. https://doi.org/10.2760/167543

(4). Vázquez-Rowe, I., Golkowska, K., Lebuf, V., Vaneeckhaute, C., Michels, E., Meers, E., Benetto, E., Koster, D., 2015. Environmental Assessment of Digestate Treatment Technologies Using LCA Methodology. Waste Management. 43, 442-459. http://dx.doi.org/10.1016/j.wasman.2015.05.007

(5). Slorach, P.G., Jeswani, H.K., Guéllar-Franca, R., Azapagic, A., 2019. Environmental Sustainability of Anaerobic Digestion of Household Food Waste. J. Environ. Management. 236. 798-814. https://doi.org/10.1016/j.jenvman.2019.02.001

(6). Pasciucco, F., Francini, G., Pecorini, I., Baccioli, A., Lombardi, L., Ferrari, L., 2023. Valorization of Biogas From the Anaerobic Co-Treatment of Sewage Sludge and Organic Waste: Life Cycle Assessment and Life Cycle Costing of Different Recovery Strategies. J. Clean. Prod. 401, 136762. https://doi.org/10.1016/j.jclepro.2023.136762

(7). AIB, 2023. European Residual Mixes. Results of the Calculation of Residual Mixes for the Calendar Year 2023. Report. https://www.aib-net.org/facts/european-residual-mix

(8). Mahmood, A. and Gheewala, S.H., 2023. A Comparative Environmental Analysis of Conventional and Organic Rice Farming in Thailand in a Life Cycle Perspective Using a Stochastic Modeling Approach. Environmental Research. 235, 116670.

(9). Ciroth, A., Muller, S., Weidema, B., Lesage, P., 2018. Empirically Based Uncertainty Factors for the Pedigree Matrix in Ecoinvent. Int J Life Cycle Assess. 21. 1338–1348. https://doi.org/10.1007/s11367-013-0670-5
